# Incidence and prevalence of neurological disorders in the United Arab Emirates: a systematic review

**DOI:** 10.1186/s12883-023-03446-6

**Published:** 2023-11-03

**Authors:** Hani T. S. Benamer, Tom Loney

**Affiliations:** 1 Department of Clinical Sciences, College of Medicine, Mohammed Bin Rashid University of Medicine and Health Sciences, Dubai, United Arab Emirates; 2 Department of Basic Sciences, College of Medicine, Mohammed Bin Rashid University of Medicine and Health Sciences, Dubai, United Arab Emirates

**Keywords:** Neurological disorders, Incidence, Prevalence, UAE

## Abstract

**Background/Objectives:**

The United Arab Emirates (UAE) is a rapidly developing country. With the increase in average life-expectancy, high rates of consanguinity, and the adoption of a Western lifestyle, the burden of neurological disorders is expected to increase over the next few decades. Despite the importance of neurological disorders, there has not been a systematic review of published studies on the epidemiology of neurological disorders in the UAE.

**Methods:**

We searched for studies of incidence and/or prevalence of neurological disorders in the UAE published in English in MEDLINE, Google Scholar, Embase, and Scopus databases with no date restrictions up until 01 October 2023. Two authors independently assessed abstracts and full texts of possibly relevant papers, followed by data extraction from studies satisfying the eligibility criteria.

**Results:**

Eight articles (*N* = 2067 patients) were included, half reported incidence and prevalence of multiple sclerosis, with an average crude prevalence 56/100,000 and related demyelinating disorders. Others were related to headache, meningitis, cerebral venous thrombosis, and brain tumours.

**Conclusion:**

There is a distinct lack of data on the epidemiology of different neurological diseases in the UAE. Large population-based studies, ideally longitudinal, are required to provide accurate and reliable estimates of the incidence and prevalence of neurological disorders to help inform healthcare capacity planning.

**Supplementary Information:**

The online version contains supplementary material available at 10.1186/s12883-023-03446-6.

## Introduction

The United Arab Emirates (UAE), which is part of the Gulf region, is a constitutional federation of seven emirates: Abu Dhabi, Dubai, Sharjah, Ajman, Ras al Khaimah, Fujairah, and Umm al Quwain. The UAE has a population of ~ 9.5 million of which approximately 70% live in the emirates of Abu Dhabi and Dubai [1]. Around 90% of the population are expatriates [1]. Abu Dhabi is the largest Emirate and is home to the capital city—Abu Dhabi. Since formation in 1971, the UAE leadership have wisely invested oil revenues to accelerate the growth of the finance, healthcare, and education sectors. Consequently, the UAE population has experienced significant improvements in health and wealth leading to an increase in life expectancy and changes in the burden of disease profile, especially age-related neurological diseases [1, 2]. Currently, there is a distinct lack of population-based data on the epidemiology of important neurological diseases. The only available data for the UAE is from the Global Burden of Disease (GBD) study which estimated the age-standardised rate of disability-adjusted life years for all neurological disorders combined to be 3647 per 100,000 population in 2015 [3].The neurological-related deaths were estimated to be 160–219 per 100,000 population [3]. Despite improvements in healthcare, the burden of neurological diseases in the UAE, especially among the Emirati population, is expected to rise due to three important factors:

1. Aging population: The UAE population has a young age structure with only 1.7% of the population aged > 65 years. However, the proportion of the population, especially amongst Emiratis, aged > 65 years is expected to increase as the life expectancy has risen over the last few decades to 78 years [1]. This demographic change will increase the burden of age-related neurological diseases such as stroke, Parkinson’s disease, and Alzheimer’s disease.
2. Genetic disorders: Consanguineous marriage, defined as marriage between relatives, leads to a higher rate of autosomal recessive disorders. The rate of consanguineous marriage amongst Emiratis in the UAE is approximately 50.5% [4]. The level of consanguinity was higher in the city of Al Ain in Abu Dhabi Emirate (54.2%) than in Dubai (40.0%) [4]. The commonest type of consanguineous marriage was between first cousins (26.2%) [4] whereas double first cousin marriages were less common (3.5%) [4].
3. Changing lifestyles: The rapid economic changes across the UAE led to increased rates of urbanisation and the adoption of Western lifestyles, including excessive caloric intake, an unhealthy diet, and a lack of physical activity [2]. This trend has led to increases in metabolic risk factors such as excess adiposity, hyperglycaemia, and hypertension [5, 6] and consequently a high prevalence of diabetes amongst the expatriate and Emirati populations [7–9]. In 2021, the estimated age-adjusted prevalence of diabetes in adults (20–79 years) in the UAE was ≥ 12% [10]. These changes will most likely have a great effect on increasing the burden of neurological diseases such as stroke and dementia.

To the best of our knowledge, this study represents the first systematic evaluation of published studies on the incidence and the prevalence of neurological disorders in the UAE.

## Methods

This paper has been written in accordance with the Preferred Reporting Items for Systematic reviews and Meta-Analyses (PRISMA) guidelines [11]. The study authors followed the standardised review methodology used in previous systematic reviews [11].

### Study design

A systematic review was conducted on epidemiological studies of neurological disease in the UAE according to the PRISMA guidelines.

### Eligibility Criteria

Studies were included if they: (1) were published in English (or an English translation was available) with no date restrictions; (2) contained incidence and/or prevalence data on neurological diseases in the UAE population. Studies that did not meet both criteria were excluded. No exclusions for age, sex, or setting were applied. Both cross-sectional and longitudinal studies were eligible for inclusion.

### Information Sources and Literature Search

The literature search was performed in MEDLINE, Google Scholar, Embase, and Scopus and the reference lists of each retrieved paper were searched manually for additional studies. We conducted a manual search by the authors’ name of all retrieved studies to identify additional studies. In collaboration with a research librarian, we conducted a broad literature search of potentially relevant papers (published on or before October 1st, 2023) on MEDLINE, Google Scholar, Embase, and Scopus using a combination of MeSH search terms and keywords to minimize the likelihood of missing evidence: related studies were conducted using ‘United Arab Emirates’ combined with ‘incidence’, ‘prevalence’, ‘epidemiology’, ‘neurology’, ‘neurological diseases’, ‘neurological disorders’, ‘stroke’, ‘epilepsy’, ‘headache’, ‘multiple sclerosis’, ‘Parkinson’s disease', and ‘dementia’ as query terms. The title and abstract of studies or the full text if necessary was reviewed to identify all relevant publications. In addition, the references in all relevant papers were reviewed for any additional publications. Only studies with data on the incidence and/or prevalence of neurological disorders published and written in English were included. We also searched Internet search engines and hand-searched the reference lists of previous systematic reviews on the topic and of all included studies.

### Study Selection

Two independent reviewers (HB and TL) manually screened the titles and abstracts of studies retrieved from the search and removed duplicates. Studies considered eligible for full text screening were retrieved for full text review. Any conflicts were resolved by discussion between the two researchers.

### Data Collection Process and Data Items

Data from each paper satisfying the inclusion criteria was extracted manually by one reviewer (HB) into a pre-defined Excel file. One reviewer (TL) independently double-checked the accuracy of the data extracted that was entered into a summary table in the following categories: Study, Neurological Disorder, Studied Population (N; % male; mean ± SD age, years), Data Collection Year(s), Diagnostic Criteria, Study Design and Setting, Prevalence (per 100,000; 95% confidence intervals), and Incidence (per 100,000 per year; 95% confidence intervals).

### Risk of Bias in Individual Studies

Both authors independently assessed the risk of bias of included studies using an adapted and modified version of the Newcastle–Ottawa Scale (NOS) [12] or observational studies specific to the context of this review (Supplementary File 1). The NOS was adapted and modified to assess the risk of bias in cross-sectional studies and retrospective chart reviews that were estimating prevalence and/or incidence for neurological diseases. Specifically, the NOS assessed the risk of bias across six domains (selection bias, sample size, outcome ascertainment, denominator bias, missing data/exclusion, statistical methods) on a three-point Likert scale 1 = ‘Low Risk’, 2 = ‘Moderate Risk’, and 3 = ‘High Risk’. The adapted and modified tool provided specific descriptions and examples of low, moderate, and high risk for each domain. The modified and adapted NOS was used to evaluate the potential risk of bias within and across studies for different domains to develop a general conclusion about the possible sources of bias on a per study and per domain basis for the included studies. Discrepancies in risk of bias domain rating between assessors were resolved through discussion.

### Summary Measures

This systematic review conducted a narrative synthesis of the included studies and reported the estimated prevalence and/or incidence of neurological diseases by study in the total UAE population and amongst expatriate and Emirati sub-populations (when available).

## Results

**Fig. 1:**
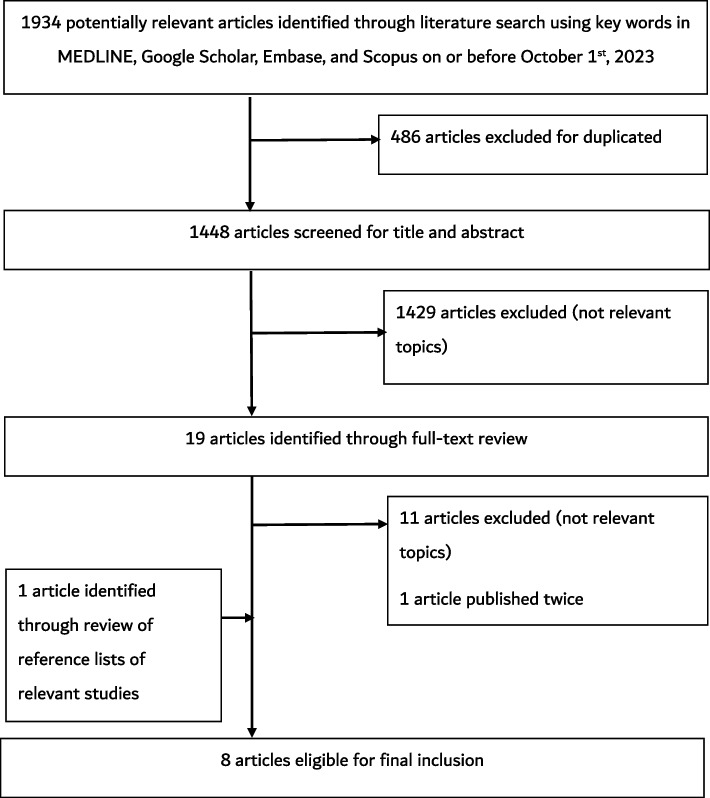
Literature search flowchart

Eight articles satisfied the inclusion criteria (Fig. 1) with a total sample size of 2067 patients with neurological diseases from 1984 to 2017 (Table 1). Six studies were retrospective [13–18] with two designed as cross sectional studies [19, 20]. Half of the articles reported incidence and prevalence of multiple sclerosis (MS) and related demyelinating disorders [13–16]. All the MS studies used the McDonald criteria as the diagnostic tool [13–15]. Other studies were related to headache [20], meningitis [17], cerebral venous thrombosis [18], and brain tumours [19]. Two of the studies reported only paediatric data [15, 20].

**Table 1:** Summary of incidence and prevalence studies of various neurological disorders in United Arab Emirates

Study	Neurological disorder	Studied population, N (% male), mean ± SD age years	Data Collection Year(s)	Diagnostic criteria	Study design and setting	Prevalence /100,000 (95% ^a^CI)		Incidence /100,000/year (95% CI)		
						**Emiratis**	**All**	**Emiratis**	**Expatriates**	**All**
Inshasi & Thakre [13]	Multiple sclerosis	Dubai, *N* = 284 (33.1%), 34.5 ± 9.9 years	2000–2007	McDonald criteria	Retrospective, single hospital	54.7 (47.0–62.6)	19.2 (13.4–25.0)	6.8 (3.8–9.8)	-	-
Schiess et al. [14]	Multiple sclerosis	Abu Dhabi Emirate, *N* = 510 (36.1%), 36.0 ± 11.0 years	2010–2014	McDonald criteria	Retrospective, 4 hospitals	57.1 (50.7–64.1); M 38.4 (31.2–46.8; F 76.9 (66.3–88.7) ^b^64.4 (57–72)	18.0 (10.0–30.0)	^b^6.0 (5.5–6.5)	-	-
Ismail et al. [15]	Multiple sclerosis	Abu Dhabi Emirate, *N* = 82 (35.0%), 7–19 years	2010–2014	McDonald criteria	Retrospective, 4 hospitals	^C^30.7 (17.5–49.9)	-	^c^2.3(0.9–4.9); M 0.7 (0.1–4.1); F 3.9 (1.3–9.2) ^d^7.2(4.3–11.4); M 7.0 (3.2–13.3); F 7.4 (3.4–14.0)	-	-
Holroyd et al. [16]	Transverse myelitis	Abu Dhabi Emirate, *N* = 46 (46.0%), 42 ± 17.6 years	2010–2016	2002 diagnostic criteria from the Consortium Working Group	Retrospective, 4 hospitals	^e^2.46	1.00	^e^0.57 f0.18	-	0.18
	Neuromyelitis optica spectrum disorders			2015 international consensus diagnostic criteria		^e^1.76	0.34	^e^0.2 ^f^0.06	-	0.05
Khan et al. [19]	Brain Tumours	United Arab Emirates, *N* = 756 (62.3%), 33.5 ± 21.1 years	1984–2017	World Health Organisation 2016 classification of brain tumours	A cross sectional study, Tawam Hospital (United Arab Emirates) Cancer Registry, large public tertiary care hospital	-	-	1.1 (0.8–1.3)	0.4 (0.3–0.4)	0.6
Dash et al. [17]	Meningitis	Al Ain District (Abu Dhabi Emirate), *N* = 92 (sex % NR), 19.0 ± 1.7 years	2000–2005	Clinical assessment, bacterial meningitis was confirmed by a positive Gram stain of cerebrospinal fluid (CSF), by cultural isolation of a relevant microorganism from CSF and/or blood, or by detection of bacterial antigens in CSF; viral meningitis was diagnosed presumptively on clinical grounds and by exclusion of bacterial meningitis	Retrospective chart review of clinical records and notification forms for cases of meningitis reported to the Department of Preventive Medicine from healthcare facilities in the medical district of Al Ain	-	-	-	-	1.0 (2000) and 2.2 (2005)
Sarathchandran et al. [18]	Cerebral venous thrombosis	Dubai, *N* = 138 (53.6%), 37.5 ± 11.3 years	2010–2018	Confirmed venous sinus occlusion on CT venograms or MR venograms	Retrospectively reviewed the medical records of all patients aged > 13 years from three major hospitals with an acute stroke service in Dubai	-	-	-	-	6.6
Bener et al. [20]	Headache	Al Ain City (Abu Dhabi Emirate), Dubai, and Sharjah emirates, *N* = 1159 (47.8%), Mean age NR	1995–1996	The diagnostic criteria defined by the International Headache Society	A cross-sectional population study, screening questionnaires followed by clinical interviews, 12 primary schools in Abu Dhabi, Dubai, and Sharjah Emirates	-	36.9%	-	-	-
	Migraine					-	13.7%	-	-	-

^a^*CI* Confidence interval, ^b^Age-standardised, ^c^Age 15–19, ^d^Age 10–14, ^e^Age ≥ 20, ^f^Age ≤ 19; NR, Not Reported

All the studies are summarised in Table 1.

### Risk of Bias Within Studies

Only three studies (*N* = 3) were rated low risk of selection bias and two studies were rated low risk of bias for sample size. The majority of studies (six) were rated low risk for outcome ascertainment bias. Only one study was rated low risk for denominator bias with five and two studies rated as moderate and high risk, respectively. Six studies were rated moderate risk for missing data/exclusion and three studies were rated as low risk for statistical analysis (Supplementary Table 1).

## Discussion

This study is the first systematic evaluation of the epidemiology of neurological diseases in the UAE. The review found limited data on the incidence and prevalence of various neurological disorders in UAE. There is no data available on some of the major neurological disorders such as arterial stroke, dementia, epilepsy, motor neuron disease, myasthenia gravis or Parkinson’s disease. Most of the studies are retrospective, hospital based, and two studies looked specifically at paediatric populations.

Two studies reported the incidence and prevalence of MS in the UAE population mainly in the Emirati population [13, 14]. One study from Dubai and the other from Abu Dhabi Emirate and both showed an MS incidence of 6–6.8 per 100,000 per year in the Emirati population [13, 14]. The reported incidence of MS in Emiratis was higher than the overall estimated incidence in the Gulf region (defined as the region which includes the following 8 countries: Bahrain, Iran, Iraq, Kuwait, Oman, Qatar, Saudi Arabia, and the UAE) of 5 per 100,000 per year [21]. The prevalence of MS in Dubai (54.7 per 100,000) and Abu Dhabi (57.1 per 100,000 and 64.4 per 100,000 when standardised for age) in the Emirati population was higher than the overall prevalence in the Gulf region (39.3 per 100,000) [21] and in the Arab countries (3.4–42 per 100,000) [22] but within the reported MS prevalence from the GBD MS study and the MS Atlas [23, 24]. This could be due to better MS surveillance or detection in the UAE compared to some other Arab countries. Furthermore, the prevalence of MS among the Emirati population was higher than median prevalence of the estimated global prevalence of 30 per 100,000 but lower than the prevalence in North American and some parts of North Europe [25]. Several environmental risk factors was proposed as possible explanations to the relatively high incidence and prevalence of MS in the Gulf region which includes; vitamin D deficiency, obesity, smoking, Epstein-Barr virus, and lifestyle modernisation [20]. However, comparing incidence and prevalence between countries and even within a country, especially over time, has limitations [20, 21]. Factors contributing to this variation include evolving diagnostic criteria, different methodology, standard and accessibility to health care services, and referral bias [22]. Further, population-based longitudinal epidemiological studies in the UAE are needed to address the question of whether there is increase in the incidence and prevalence of MS which has been reported in other countries and regions in the world [22, 26–28].

Dash et al. reported reduced incidence of meningitis in Al-Ain district in UAE from 2000–2005 with a significant reduction in the incidence of *Haemophilus influenzae* type b after the introduction of the national immunisation programme [17]. However, they concluded that *“Improved methods of bacterial detection including isolate serotyping must be made available in order to further reduce mortality and morbidity from meningitis* [17]*”.* No further studies were conducted to assess whether there has been further reduction in the incidence, morbidity, and mortality from meningitis. Therefore, there is an urgent need for continued research to assess the temporal changes in the burden and mortality associated with meningitis in the UAE.

Our review found only one study related to cerebrovascular diseases. A retrospective review of medical records in a single hospital in Dubai showed an average frequency of 6.6 cerebral venous thrombosis per 100,000 population [18]. The frequency increased during the hottest months of the year probably due to dehydration [18]. Anaemia and polycythaemia were strongly associated with a thrombotic event [18]. The authors suggested that the reported incidence rate was lower than population-based studies reported elsewhere and suggested that larger longitudinal studies are needed [18]. The only data on arterial stroke from UAE was published in the GBD Stroke study which showed an incidence of all strokes in the UAE at ≥ 218.3 per 100,000 population which represent the highest global rate [29]. The incidence of ischaemic stroke was reported by the same study at ≥ 15.5 per 100,000 population which again represents the highest global rate [29]. On the contrary the incidence of intracerebral haemorrhage and subarachnoid haemorrhage was reported as 39.7- < 48.6 and 11- < 13.3 per 100,000 population respectively which is on the lower side in comparison with other countries [29].These results should be used as guidance when planning further studies in the UAE, for example exploring the reasons leading to the high incidence of ischaemic stroke, with low rate of haemorrhagic stroke in comparison with other countries. We are of the opinion that urgent arterial stroke epidemiological studies are needed to address such an important preventable disease.

The analysis of 756 cases with primary malignant brain tumour using the hospital cancer registry at Tawam hospital, in Al Ain city (the first cancer referral hospital in the UAE), showed the highest incidence rate was in 2008 (1.04 per 100,000) with declining rates thereafter (0.40 per 100,000 in 2017) [19]. A rapid increase in the UAE expatriate population and the creation of another cancer registry in Dubai were suggested as possible explanations suggesting that changing population dynamics and increased detection might account for the observed changes [19]. Establishing a UAE-wide registry with a clear differentiation between the Emirati and expatriate populations will help in understanding the brain tumour incidence trend in the UAE.

The population structure of the UAE is unusual but has some similarity with other Arab Gulf countries (Bahrain, Kuwait, Oman, Qatar, and Saudi Arabia) where expatriates constitute a significant portion of the population (approximately 90% of the UAE population are expatriates [2]). Most expatriates are young males from South Asian countries working in the construction sector or other physically demanding occupations. These jobs require a certain level of health and occupational fitness which could lead to a “healthy worker effect” and the prevalence or incidence of neurological diseases is likely to be lower amongst expatriates compared to the same aged adults in their home countries. Also, the expatriate population are highly mobile and likely to return to their home country once they are diagnosed with a chronic illness to seek treatment due to accessibility and/or cost of the healthcare services in the UAE. Epidemiological data from Emirati and expatriate populations, taking in consideration the structure of the population, will help in planning and delivering health services. However, detailed epidemiological studies of the Emirati population, which are young and relatively static and homogenous, will add to the body of knowledge and help in understanding the epidemiology of neurological disorders including the incidence, prevalence, clinical presentation, disability and quality of life prognosis, survival, and treatment outcomes.

The only available epidemiological data on headache, epilepsy and Parkinson’s disease is published by various GBD studies [30–32]. For epilepsy the estimated age-standardised prevalence was report at 380- < 430 per 100,000 population in 2016 [30], and 100- < 110 for Parkinson’s disease [32]. Both rates are in the middle range in comparison with other countries [30, 32]. For migraine and tension headache the estimated age-standardised prevalence was report at 13,000- < 14,000 and 25,000- < 27,000 per 100,000 population in 2016, respectively [31]. Again, both rates are in the middle range when compared with other countries [31]. There are several limitations in the GBD studies, such as*, “original epidemiological data were not available for all countries”* [30]*, and “Methodological differences for determining prevalence and study shortcomings might result in estimates that vary considerably”* [32]*.* However, such data can be used as guidance to the burden of various neurological disorders in the UAE until further epidemiological studies take place as original data from the UAE is missing.

The risk of bias assessment highlighted several common methodological limitations in the eight included studies. Firstly, the use of cross-sectional or retrospective chart review study designs that did not collect or have access to important socio-demographic data that might be useful to healthcare planners. Secondly, recruitment of patients or use of data from single sites or single emirates that might create a selection bias whereby the included patients do not reflect all patients in the UAE with a specific neurological disease. Finally, the population data used for the denominator in the calculation of prevalence/incidence estimates was not from fully enumerated census data at the emirate or country level and/or from a different time period to the case recruitment/diagnosis. As such, this review has highlighted the need for well-designed, adequately powered prospective studies in order to provide accurate and reliable incidence estimates of important neurological diseases in the UAE. One cost-effective approach would be to develop neurological disease registries with data linkage to nationally representative census, education, and employment databases.

In conclusion, this systematic review shows a clear deficit in incidence and prevalence data regarding different neurological diseases in the UAE. Consequently, it is not possible to provide an accurate and reliable estimate of the burden of neurological disorders in the country. Therefore, there are significant opportunities for further well-designed prospective communicate-based epidemiological studies on a larger scale than currently reported in the UAE. National registries of major neurological diseases such as stroke, epilepsy, MS, and Parkinson’s disease for the Emirati population could be the first step to establish proper epidemiological research. Such research would enhance our understanding and potentially the management of major neurological disorders in UAE, the Gulf region, Arab countries, and globally.

### Supplementary Information

**Additional file 1:** **Supplementary File 1. **Modified Newcastle-Ottawa quality assessment scale (adapted for cross-sectional studies and retrospective chart reviews).

**Additional file 2:** **Supplementary Table 1. **Newcastle-Ottawa risk of bias assessment for included studies.

## Data Availability

The datasets used and/or analysed during the current study available from the corresponding author on reasonable request.
