# Supplementary material for: Incidence and prevalence of neurological disorders in the United Arab Emirates: a systematic review
Source: BMC Neurol. 2023 Nov 3;23:396. doi: 10.1186/s12883-023-03446-6 (PMC10623824; doi:10.1186/s12883-023-03446-6)
Supplement: Supplementary file 1 — Additional file 1: Supplementary File 1. Modified Newcastle-Ottawa quality assessment scale (adapted for cross-sectional studies and retrospective chart reviews). [file 12883_2023_3446_MOESM1_ESM.docx]

**Supplementary File 1 – Modified Newcastle-Ottawa Quality Assessment Scale (adapted for cross-sectional studies and retrospective chart reviews)**

Adapted and modified from Perera, S. et al. Association between body mass index and suicidal behaviors: a systematic review protocol. Systematic Reviews;2015: 4:52. DOI 10.1186/s13643-015-0038-y.

**Domain: Selection Bias (maximum score 3)**

**Was the source population appropriate and representative of the population of interest?**

(rated from 1 ‘Low Risk’ of bias to 2 ‘Moderate Risk’ of bias to 3 ‘High Risk’ of bias)

*Example of low risk of bias:* A consecutive sample or random selection from a population that is representative of the condition under study.

*Example of moderate risk of bias:* A consecutive sample or random selection from a population that is not highly representative of the outcome of interest.

*Example of high risk of bias:* The source population cannot be defined or enumerated (i.e.

volunteering or self-recruitment).

**Domain: Sample Size (maximum score 3)**

(rated from 1 ‘Low Risk’ of bias to 2 ‘Moderate Risk’ of bias to 3 ‘High Risk’ of bias)

**Was the sample size sufficient and was there sufficient power to detect a meaningful difference in the exposure/outcome of interest?**

*Example of low risk of bias:* Sample size was adequate and there was sufficient power to detect a difference in the exposure/outcome; or the sample included all available cases in time-frame sample.

*Example of moderate risk of bias:* Sample size calculation was reported but target sample was not achieved due to non-response, participation rates, or missing data.

*Example of high risk of bias:* Sample size was small and there was not sufficient power to test the outcome of interest; or a sample size calculation was not reported; or the sample did not include all available cases within the recruitment time frame and justification was not provided for exclusion.

**Domain: Outcome Ascertainment (maximum score 3)**

**Was the outcome ascertainment method appropriate?**

(rated from 1 ‘Low Risk’ of bias to 2 ‘Moderate Risk’ of bias to 3 ‘High Risk’ of bias)

*Example of low risk of bias:* The study used the appropriate/gold standard diagnostic method and criteria.

*Example of moderate risk bias:* The study used subjective/self-reported methods to ascertain outcome

Example of high risk of bias: The study provided limited information on the methods of

measuring the outcome and the measure was not appropriate considering the outcome.

**Domain: Denominator Bias (maximum score 3)**

**Did the study use accurate and reliable population estimates for the denominator?**

(rated from 1 ‘Low Risk’ of bias to 2 ‘Moderate Risk’ of bias to 3 ‘High Risk’ of bias)

*Example of low risk of bias:* The study used population estimates from fully enumerated census data for the whole sample and sub-groups from the same time period as case recruitment.

*Example of moderate risk bias:* The study used population estimates from fully enumerated census data from an earlier time period compared to case recruitment; or partially enumerated population estimates from the same time period as case recruitment; or extrapolated population estimates using an appropriate method.

*Example of high risk of bias:* The study did not use appropriate population estimates for the denominator; or the source of the denominator population estimates were not stated.

**Domain: Missing Data/Exclusion (maximum score 3)**

**Was there minimal missing data and did the study handle it accordingly?**

(rated from 1 ‘Low Risk’ of bias to 2 ‘Moderate Risk’ of bias to 3 ‘High Risk’ of bias)

*Example of low risk of bias:* The study reported missing data to be less than 10% with reasons and specified the method of handling it; or valid reasons for exclusion were reported.

*Examples of moderate risk bias:* The study reported greater than 10% missing data but specified the method used to handle it; or reasons for exclusion were reported but not justified.

*Example of high risk of bias:* The study had greater than 10% missing data and did not provide reasons or the methods used to handle it or the study did not report on missing data/reasons for exclusion.

**Domain: Statistical Methods (maximum score 3)**

**Did the study use appropriate statistical analysis methods relative to the study objectives and outcome of interest?**

(rated from 1 ‘Low Risk’ of bias to 2 ‘Moderate Risk’ of bias to 3 ‘High Risk’ of bias)

*Example of low risk of bias:* The study reported use of appropriate statistical analysis as required.

*Examples of moderate risk bias:* The study used correct statistical methods but did not report them in sufficient detail, or used the incorrect methods but reported them in detail.

*Example of high risk of bias:*
