# Supplementary material for: Incidence and prevalence of neurological disorders in the United Arab Emirates: a systematic review
Source: BMC Neurol. 2023 Nov 3;23:396. doi: 10.1186/s12883-023-03446-6 (PMC10623824; doi:10.1186/s12883-023-03446-6)
Supplement: Supplementary file 2 — Additional file 2: Supplementary Table 1. Newcastle-Ottawa risk of bias assessment for included studies. [file 12883_2023_3446_MOESM2_ESM.docx]

**Supplementary Table 1. Newcastle-Ottawa Risk of Bias Assessment for Included Studies**

| **Domain** | **Selection Bias** | | **Sample Size** | | **Outcome Ascertainment** | | **Denominator Bias** | | **Missing Data/Exclusion** | | **Statistical Methods** | |
| --- | --- | --- | --- | --- | --- | --- | --- | --- | --- | --- | --- | --- |
| **Study** | **Score (1-3)** | **Justification** | **Score (1-3)** | **Justification** | **Score (1-3)** | **Justification** | **Score (1-3)** | **Justification** | **Score (1-3)** | **Justification** | **Score (1-3)** | **Justification** |
| **Inshasi & Thakre [12]** | **3 (High Risk)** | Retrospective, single government major tertiary referral hospital in Dubai emirate 2000-07 | **2 (Mod Risk)** | N=284 (56% Emirati) – unclear whether sample included all available cases within timeframe | **1 (Low Risk)** | McDonald’s criteria based on medical records | **2 (Mod Risk)** | 2006 Dubai and UAE census estimates for UAE nationals and expatriates | **2 (Mod Risk)** | Valid reasons for case exclusion reported. Missing data not reported | **3 (High Risk)** | Crude prevalence estimates and incidence rates reported not accounting for age-structure of sub-groups |
| **Schiess et al [13]** | **2 (Mod Risk)** | Retrospective, four largest government hospitals in Abu Dhabi emirate 2010-14 | **2 (Mod Risk)** | N=510 (62% Emirati) - complete case ascertainment for UAE nationals but probably not expatriates | **1 (Low Risk)** | ICD9 codes from medical records reviewed by MS neurologist – 2011 McDonald’s criteria | **2 (Mod Risk)** | Age-group and sex specific MS prevalence for Emirati nationals resident in Abu Dhabi were calculated using total number of reports as the numerator and the 2014 mid-year population as the denominator | **2 (Mod Risk)** | Valid reasons for case exclusion reported. Missing data not reported | **1 (Low Risk)** | Standardization was carried out using the WHO World Standard Population. Diagnosed cases were the numerator and mid-year population as the denominator both summed over the five years 2010–2014 |
| **Ismail et al [14]** | **2 (Mod Risk)** | Retrospective, four largest hospitals in Abu Dhabi emirate 2010-14 | **2 (Mod Risk)** | N=82 (65% Emirati) - complete case ascertainment for UAE nationals but probably not expatriates | **1 (Low Risk)** | ICD9 codes from medical records reviewed by MS neurologist – 2010 McDonald’s criteria | **2 (Mod Risk)** | Denominator for calculating prevalence (incidence) was the population of Abu Dhabi (in appropriate  5-year age groups estimated at December 2014) as the average of the mid-year populations of 2014 and 2015 (sum of the midyear populations  2010, 2011, 2012, 2013, and 2014 for incidence) | **2 (Mod Risk)** | Valid reasons for case exclusion reported. Nine (11%) cases with missing clinical data | **1 (Low Risk)** | Age- and sex-adjusted prevalence using population denominator data in 5-year age groups 0-4, 5-9, 10-14, and 15-19. Exact 95% confidence intervals were calculated using the Poisson distribution |
| **Holroyd et al [15]** | **2 (Mod Risk)** | Retrospective, four large government hospitals in Abu Dhabi emirate 2010-16 | **2 (Mod Risk)** | N=46 (50% Emirati) - complete case ascertainment for UAE nationals but probably not expatriates | **2 (Mod Risk)** | ICD9 codes from medical records medical records reviewed by neurologist. Transverse myelitis and neuromyelitis optica spectrum disorders (NMSOD) were defined using the 2002 and 2010 (respectively) diagnostic criteria from the Consortium Working Groups; 44% of patients in were not tested for AQP4 IgG or MOG IgG antibodies - underestimation of NMOSD prevalence | **2 (Mod Risk)** | Estimate of total mid-year population of adult Emiratis in Abu Dhabi in 2016 | **2 (Mod Risk)** | Valid reasons for case exclusion reported. Level of note detail and tests and imaging ordered varied greatly between  providers. Missing data not quantified | **2 (Mod Risk)** | Crude prevalence with age adjustment by subtracting estimated number of children to calculate Emirati adult population |
| **Khan et al [18]** | **2 (Mod Risk)** | Retrospective, single large government hospital in Al Ain city (Abu Dhabi emirate) with cancer registry data for all cases of brain cancer in the UAE from 1984-2017 | **2 (Mod Risk)** | N=756 (28% Emirati) - complete case ascertainment for UAE nationals but probably not expatriates | **1 (Low Risk)** | ICD-O-3 used to define cases and tumour histological codes were matched and grouped using the WHO 2016 classification | **3 (High Risk)** | Population data for calculations was acquired from the  World Bank data and Central Intelligence Fact Sheet. | **2 (Mod Risk)** | Data obtained from a 33-year period with varying data collection protocols leading to missing data for several data categories. Tumours with missing histologic confirmation  or not classified under the ICD-O-3 site codes were excluded. Restricted dataset used when nationality and Emirate of residence was missing | **2 (Mod Risk)** | Average annual crude incidence rates and 95% confidence intervals were estimated per  100,000 population, based on one-year age groupings. Average annual crude incidence rates age-adjusted to  the 2017 Gulf Cooperation Council (GCC) population  and 2000 United States (US) standard population. Direct method of standardization of  was applied for years 2013-2017 |
| **Dash et al [16]** | **1 (Low Risk)** | Retrospective – Preventive Medicine Department in one city of Abu Dhabi emirate 2000 to mid-2005 | **1 (Low Risk)** | N=92 – mandatory notifiable disease with all cases reported to Preventive Medicine Department within 24 hours | **2 (Mod Risk)** | Case definitions: (1) meningitis diagnosis based on clinical assessment; (2) bacterial meningitis confirmed by a positive Gram stain of cerebrospinal fluid (CSF), by cultural isolation of a relevant microorganism from CSF and/or blood, or by detection of  bacterial antigens in CSF; (3) viral meningitis diagnosed  presumptively on clinical grounds and by exclusion of bacterial meningitis. Nine (10%) cases of  undetermined etiology and failure  to isolate a causative microorganism from 19 (33%) cases. | **3 (High Risk)** | Unclear which population estimates for Al Ain city were used as denominator for calculating the incidence rates. | **2 (Mod Risk)** | Vaccination status was not available for four cases (8%) of meningitis due to Mycobacterium tuberculosis. | **3 (High Risk)** | The study did not report on the statistical methods used. |
| **Sarathchandran et al [17]** | **1 (Low Risk)** | Retrospective, consecutive cases from three major hospitals with an acute stroke service in Dubai emirate 2010-18 | **1 (Low Risk)** | N=138 (55% South Asian suggestive of a representative sample), consecutive time frame sample from four major hospitals | **1 (Low Risk)** | ICD9 codes from medical records and only cases with confirmed venous sinus occlusion on  CT venograms or MR venograms were included | **2 (Mod Risk)** | Average adult population estimates of Dubai from January 2010 to December 2018 obtained from Dubai Statistics Centre (Government of Dubai). | **3 (High Risk)** | Study did not report excluded cases or missing data. | **2 (Mod Risk)** | Study reported use of appropriate statistical analysis but not calculation of crude incidence rates. |
| **Bener et al [19]** | **1 (Low Risk)** | Cross-sectional, multistage stratified cluster sampling to select 12 schools with UAE national children from Al Ain city (Abu Dhabi Emirate), Dubai emirate, and Sharjah emirate Oct 1995 to Jun 1996 – UAE nationals only (expatriate children not included) | **2 (Mod Risk)** | N=1159 (aged 6-14 years; 83% participation rate) random representative sample of UAE nationals. Power calculation estimated sample N=1400 required. | **1 (Low Risk)** | Forward and back translation (English-Arabic) of standardised questionnaire to diagnose headache and migraine by International Headache Society followed by standardized clinical interview with physicians and qualified nurses in their mother tongue with physical and neurological examinations. | **1 (Low Risk)** | Multistage stratified cluster sampling to select representative sample of UAE nationals (expatriate children not included) | **1 (Low Risk)** | N=241 students excluded for incomplete questionnaires or school absence (83% participation rate) | **1 (Low Risk)** | Study reported use of appropriate statistical analysis. |

*Note.* Score 1 denotes Low Risk of Bias, 2 denotes Moderate Risk of Bias, 3 denotes High Risk of Bias.
